# Analysis of chromosomal aberration (1, 3, and 8) and association of microRNAs in uveal melanoma

**Published:** 2009-10-22

**Authors:** Abhirami Radhakrishnan, Nirmala Badhrinarayanan, Jyotirmay Biswas, Subramanian Krishnakumar

**Affiliations:** L&T Department of Ocular Pathology, Vision Research Foundation, Sankara Nethralaya, Chennai, India

## Abstract

**Purpose:**

Uveal melanoma is the most common intraocular primary tumor, involving iris, ciliary body and choroid. More than 90% of the patients develop hepatic metastasis with an average survival time of 7 months. We have used formalin fixed paraffin embedded sections to validate the presence of monosomy 3, an accurate predictor of metastasis, chromosome 8 isochromosome (8q22), and 1p36 deletion. This study also tested the presence of oncomirs in uveal melanoma samples by microRNA (miRNA) expression profiling.

**Methods:**

Chromogenic in situ hybridization (CISH) was performed in formalin fixed, paraffin embedded sections of uveal melanoma to analyze chromosome 1, 3, and 8 aberrations (n=60). MicroRNA (miRNA) expression profiling was done on paraffin sections of invasive tumor with liver metastasis (n=1) and non invasive tumor (n=1) in biological duplicates. Samples for miRNA expression profiling were identified based on case registry and the harboring of monosomy 3 by CISH.

**Results:**

A significant correlation (p=0.05) between metastasizing and non-metastasizing melanoma harboring chromosomal aberrations- monosomy3, c-myc, and 1p36 was observed by Pearson’s correlation. A significant correlation was observed in monosomy 3 and 1p36 positive cases in the tumor samples (p=0.039). No significance was observed between monosomy 3 and c-myc positive cases. No significance (p=0.096) was observed between c-myc amplification and trisomy (extra whole chromosome 8). MicroRNA expression profiling revealed the presence of 19 miRNAs expressed in non-metastasizing melanoma and absent in metastasizing melanoma. Eleven miRNAs were found to be expressed in metastasizing melanoma and absent in non-metastasizing melanoma. Genes targeted by the miRNAs were found to be present in chromosomal regions 8p22, 13q, and 17p but were often found to be deleted.

**Conclusions:**

This technique can be applied to routine pathology using archival specimens to identify patients with monosomy 3. We were able to perform CISH in all the cases except the heavily pigmented tumors, where dots were observed but were not assessed. Initial studies on microRNA have revealed their role as oncomirs in both metastasizing and non-metastasizing melanomas. Further studies may provide insights into their role in tumor progression and facilitate metastatic phenotype analysis.

## Introduction

Aneuploidy in cancer has been studied widely by cytogenetic techniques to identify molecular determinants in metastasis. Uveal melanoma is a common primary intraocular tumor, originating from melanocytes and metastasizes to the liver in 95% patients leading to death within 2-14 months [1]. This makes uveal melanoma an attractive model to study the indicators of tumor metastasis. Tumor related factors include extra ocular extension, tumor size, anterior tumor location, increased tumor invasiveness, tumor pigmentation, microvascular patterns, microvascular density and cell type. Uveal melanoma is known to carry deletions in 3p25, 3q24, all of chromosome3, 1p36, 6q23, 9p21-9p23, 13q14, 13q12.3-q13, and 17p13. Amplification observed in 1q, 16p, 20q , 22q [2,3], 8q22, and an extra chromosome 8 [4]. Conventional cytogenetics including fluorescent in situ hybridization (FISH) and Chromogenic in situ hybridization (CISH) have demonstrated that non-random chromosomal abnormalities, the loss of chromosome 3, and amplification of 8q are related to high mortality [3] and the loss of 1p36 in metastasizing tumors is associated with concurrent monosomy 3 [5]. Aberrations in 6p and 6q regions have been observed to be involved in tumor initiation and development rather than tumor progression [6]. Sandinha et al. [7] has identified monosomy 3 in metastasizing melanoma by CISH.

CISH is being used to study many cancers as a molecular and cytogenetic tool to assess tumorogenicity, becoming an important component in molecular diagnostics and research studies. This technique is generally monochromatic, allowing a single target sequence to be detected using alkaline phosphatase/peroxidase reactions. The signals and tissue morphology can be simultaneously detected by bright field microscopy. Recent advances in this technique has led to dual color CISH, which has enabled aberrations in breast carcinoma to be studied by detection of biotin and digoxigenin labeled probes simultaneously within the nuclei, resulting in green and red chromogenic signals. A ratio of centromere to other chromosomal regions is studied to visualize relevant diagnostic translocations in the tumor sample, resulting in direct correlation of genotypic and phenotypic characteristics.

Human epidermal growth factor receptor-2 amplification analysis by CISH in breast cancer has revealed highly reproducible results concordant with Fluorescent in situ hybridization and immunohistochemistry and as a viable alternative to the other techniques [8,9]. Cytological smears are also found to be good source to study Her-2/neu status in breast cancer by CISH. Other cancers such as squamous cell esophageal carcinoma [10] and non-small cell lung carcinoma [11] have been studied for their respective chromosomal aberration in individual cancer by CISH.

Advent of gene expression and miRNA profiling in uveal melanoma has led to the identification of molecular markers, that classify tumors as Class I (low metastatic risk) and Class II (high metastatic risk). Studies on gene expression profiling have identified downregulated genes in chromosome 3p region [12] and upregulated regions in chromosome 8q [13]. *LZTS1*, a tumor suppressor gene was found to be deleted in 8p22 region [14]. A study reports amplification of *DDEF1* located in 8q24 [15]. miRNA expression profiling has identified significant miRNA discriminators in class I and Class II and specific miRNA associated with metastatic priority [16].

## Methods

### Study population

The study was reviewed and approved by the local ethics committee the study conformed to the generally accepted principles of research in accordance with the Helsinki Declaration. Archival specimens of choroidal melanoma were identified from the patient medical records from 1994-2009 (15 years).The tumors were categorized as invasive (with liver metastasis), non-invasive, intrascleral, or extrascleral. Patients with liver metastasis were confirmed by case records including imaging and liver tests. All tissues had been previously fixed in neutral buffered formalin for 12-24 h. All tumor slides stained with hematoxylin and eosin were examined for invasion of choroid, optic nerve, intra and extrascleral extension, necrosis, and pigmentation after which 1-2 representative tumor tissue blocks were selected. A total of 60 tumor samples and 11 retinas were taken for use as internal controls. For miRNA expression analysis, 2x20 µm formalin fixed, paraffin embedded sections were taken from a metastasizing tumor and non-metastasizing case identified from the case registry. Our CISH studies confirmed that the selected tumor sections harbored monosomy 3 aberration, in the metastasizing case and no aberration in the non -metastasizing case.

### Centromeric and locus specific probes

All probes used were purchased from Zymed Laboratories (San Francisco, CA). Unlike traditional cytogenetic DNA probes which require repetitive specific blocking; these probes were created by eliminating repetitive sequences using subtraction probe technology. They are doublestranded and labeled with biotin/digoxigenin (DIG). Centromeric probes labeled with biotin have been used to study monosomy in chromosome 3 and extra chromosome 8 (trisomy). Locus-specific probes used to study chromosome 8–c-myc amplification and 1p36 deletion were labeled with digoxigenin. Centromeric probe 18 was employed as the control probe as aneuploidy in chromosome 18 was not observed in choroidal melanoma.

### Tissue section preparation

Four to five μm tissue sections were placed on Super Frost Plus positively-charged slides and baked at 60 **°**C for 6-18 h. The slides were then deparaffinized in 100% xylene, rehydrated, and cleared in a graded series of methanol from 100% -60% to water.

Depigmentation was performed for moderately pigmented and heavily pigmented sections. Moderately pigmented tumors were depigmented in 0.25% potassium permanganate and 5% oxalic acid. Heavily pigmented tumors were depigmented in 3.0% (vol/vol) hydrogen peroxide and 1.0% (wt/vol) disodium hydrogen phosphate, 18 h at room temperature overnight. Sections were subjected to heat pretreatment in TRIS –EDTA, pH 8.0 at 98 °C for 15 min. After rapid cooling in water for few seconds, a PBS wash was performed. Tissues were incubated with pepsin, an enzyme pretreatment reagent (Zymed Laboratories) for 8-10 min washed in PBS followed by dehydration in a graded series of alcohol and air dried for 15-20 min.

### Denaturation and hybridization of probe

Probe in formamide hybridization mix was added to the tissue sections as per the tissue size. Cover slips were sealed over the tissue with a rubber solution to prevent evaporation during the subsequent denaturation step at 95 ºC for 6 min was carried out in a gradient in situ PCR thermal cycler followed by overnight for (18 h) hybridization.

### Immunohistochemical and signal detection of probe

Partially bound and unbound probes were removed by 0.5× SSC wash at room temperature for 5 min and 75 ºC for 5 min. Endogenous peroxidase was removed by submerging the slides in 3% hydrogen peroxidase in methanol. Slides were incubated with CAS block reagent (zymed for 10 min). For locus specific probes, slides were incubated for 1 h in mouse anti-digoxigenin antibody followed by rinse in PBS containing 0.025% Tween-20. The slides were then incubated with goat anti-mouse HRP polymer conjugate for 1 h and later rinsed in PBS-Tween. Tissues hybridized with centromeric probes were incubated with HRP-streptavidin for 1 h. Slides were further incubated with DAB substrate for 30 min. The sections were rinsed in tap water and counterstained with haematoxylin for a few seconds. The slides were then washed in running tap water, dehydrated in graded series of methanol and mounted with histomount.

### Analysis – quantification of hybridization signals

The tissue sections were examined by light microscopy using an oil immersion lens (magnification 1000×). CISH results were independently evaluated by two pathologists and were found to be reproducible. A minimum of 100 nuclei were counted. The chromosomal aberrations were assessed by using both chromosomal index and signal distribution (SD). Chromosomal index is calculated by dividing the number of hybridization sites by the number of nuclei counted, this gives the average chromosome number. The chromosome index (CI), which gives an average chromosome copy number, was calculated by dividing the total number of hybridization spots counted by the total number of nuclei counted [7].

Chromosome loss is defined as CI<3 SD from the mean for retina (normal tissue ), for chromosomes 1 and 3. Chromosome 8 amplification was defined as disomy in case of two dots, extra chromosome - trisomy and amplified (4-8 copies). SD is defined as the % of nuclei with only one hybridization in case of chromosomes 1 and 3. The tumor samples had to show chromosomal loss by both CI and SD to be regarded as monosomy or deleted [7].

### MicroRNA isolation and expression analysis

#### Sample processing and RNA extraction

Sections (2x20 µm) of metastasizing melanoma and non metastasizing melanoma were selected and deparrafinized in xylene and washed with ethanol and subjected to overnight protease digestion. Total RNA was isolated using TRI-Reagent (Ambion, Austin,TX). RNA concentration and purity was quantified using Nanodrop (Nanodrop Santa Clara, CA) spectrophotometer by nanodrop ribogreen assay and quality of total RNA was determined on an Agilent (Agilent, Santa Clara, CA) bioanalyzer.

#### Agilent arrays- hybridization and detection

Human miRNA V2 8x15k Agilent arrays which represents 723 human and 76 human viral miRNAs were used for miRNA analysis. Total RNA underwent phosphatase treatment. The 3’end of dephosphorylated RNA was ligated with one molecule of (3-pCp), cyanine 3-cytidine bisphosphate (pCp) as this reagent selectively labels and hybridizes mature miRNAs, with greater than 30% efficiency. Hybridization cocktail was added to the arrays and hybridization was performed in the hybridization oven set at 55 ºC for 20 h. The microarrays were washed using Agilent wash buffer. Scanning and extraction was performed using Agilent feature extraction software. The study was done in biological replicates. The intensities and background subtracted were taken from the Raw Data file generated from Agilent Feature Extraction software (Aligent). The Sum of background-subtracted signals for each repeated miRNA was calculated. The sum of background-subtracted signal for each of repeated miRNA was log transformed to log base 2. Cut off used was greater than 1 (Log Trasnformed value) in metastatic and less than 1(Log Trasnformed value) in non- metastatic and vice versa.

## Results

### Assessment of chromosomal index and signal distribution

**Figure 1:**
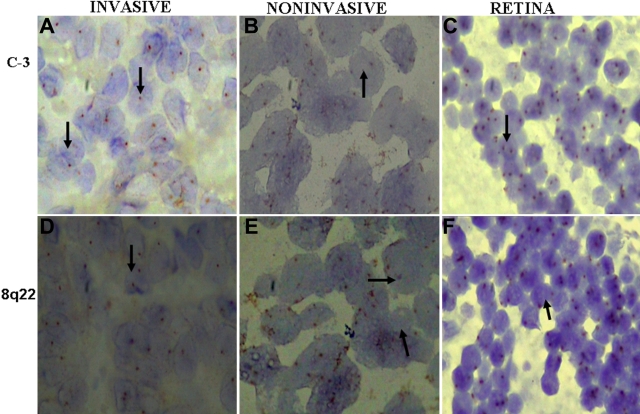
Chromogenic in situ hybridization for chromosome 3 and 8q22. **A**: Invasive melanoma, hybridized with chromosome 3 showing single copy in all the cells. **B**: Non-metastasising melanoma, hybridized with chromosome 3 showing two copies in most of the cells. **C**: Normal retina hybridized with chromosome 3 showing two copies in all the cells. **D**: Invasive melanoma, hybridized with chromosome 8q22 showing two copies in most of the cells. **E**: Non-metastasising melanoma, hybridized with chromosome 8q22 showing two copies in most of the cells. **F**: Normal retina hybridized with chromosome 8q22 showing two copies in all the cells.

**Figure 2:**
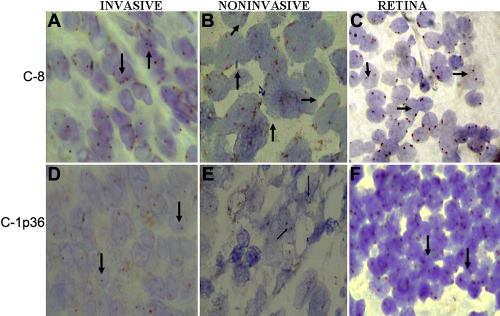
Chromosome in situ hybridization for aberrations in chromosome 8 (extra whole chromosome and chromosome 1 (1p36). **A**: Invasive melanoma, hybridized with chromosome 8 showing trisomy in few cells and two copies in most of the cells. **B**: Non-metastasising melanoma, hybridized with chromosome 8 showing two copies in most of the cells. **C**: Normal retina hybridized with chromosome 8 showing two copies in all the cells. **D**: Invasive melanoma, hybridized with chromosome 1p36 showing one copy in all the cells. **E**: Non-metastasizing melanoma, hybridized with chromosome 1p36 showing two copies in most of the cells. **F**: Normal retina hybridized with chromosome 1p36 showing two copies in all the cells.

**Figure 3:**
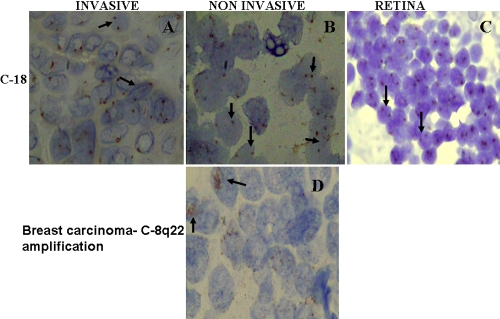
Chromogenic in situ hybridization for chromosome 18 and 8q22 amplification in breast carcinoma. **A**: Invasive melanoma, hybridized with chromosome 18 showing two copies in all the cells. **B**: Non-metastasizing melanoma hybridized with chromosome 18 showing two copies in most of the cells. **C**: Normal retina hybridized with chromosome 18 showing two copies in all the cells. **D**: Breast carcinoma hybridized with chromosome 8q22 showing positive amplification (large cluster).

In situ hybridization for chromosome 1p36, 3, 8, and 18 was successfully performed for 49 samples, 11 samples were not taken into account due to heavy pigmentation. Hybridization signals obtained as a result of addition of locus specific and centromeric probes were analyzed on the basis of chromosomal index and signal distribution. For each tumor section (n=49) number of signal spots per nucleus was counted for 100 nuclei and the pathologist was masked to the outcome of patient. Overlapping nuclei and over digested cells with poor quality hybridization signal was excluded. For chromosome 1p36 and 3 the mean CI for retina was 1.56 and 1.53. A tumor was defined as monosomic for chromosome 3 if its CI was less than 3SD from the mean. Signal distribution is the percentage of nuclei with one hybridization site greater than 30% of nuclei counted. This analysis was performed as per criteria taken in [17]. Nuclei carrying <15% of hybridized spots was not considered to be harboring the respective chromosomal aberration (Table 1). Liver metastatic case of choroidal melanoma hybridized with chromosome 3 showed a single copy in nearly all the cells, whereas in noninvasive melanoma and retina showed two copies (Figure 1). Other examples of metastatic melanoma, choroidal melanoma and normal retina hybridized with 1p36 are shown in (Figure 2D-F ), 8q22 (Figure 1D-F), whole chromosome 8 (Figure 2A-C), and chromosome 18 (Figure 3A-C). 8q22 amplification in breast carcinoma is seen as large clusters as shown in Figure 3D.

**Table 1:** Summary of signal distribution for chromosome 3, 8, and 1.

**Probe**	**<15%**	**30%**	**40%**	**50%**	**60%**	**70%**
a. Monosomy3	35	8	3	3	-	-
b. c-myc-8	43	1	-	-	2	3
c. Trisomy-8	47	-	-	1	1	-
d. Deletion-1p36	44	1	-	1	-	3

The first column a-d represents the chromosomal aberration observed in uveal melanoma samples (n=49) in the respective percentages, <15% was not taken into account for the respective aberration. Hybridization site >30% was taken as positive for signal distribution analysis.

### Statistical significance

A significant correlation(p=0.05) between metastasizing and non metastasizing uveal melanoma with respect to the chromosomal aberrations- monosomy 3,C-myc and 1p36 was observed by Pearson’s correlation. There exists a significant correlation between the two classes with respect to the balanced expression of the chromosomes. A significant correlation was observed in monosomy and 1p36 positive cases in the tumor samples (p=0.039). No significance was observed between monosomy 3 and c-myc positive cases. No significance was observed between c-myc amplification and trisomy (extra whole chromosome 8; p=0.096).

### MicroRNA expression analysis

The data discussed in this publication have been deposited in NCBI's Gene Expression Omnibus [18] and are accessible through GEO Series accession number GSE17037. Nineteen miRNAs were found to be expressed in non metastatic tumor and unexpressed in metastatic tumors. 11 miRNAs were found to be expressed in metastatic tumors and unexpressed in non metastatic tumor (Table 2 and Table 3).

**Table 2:** The list of oncomirs in uveal melanoma targeting metastasis suppressor genes.

**miRNA**	**Chromosome allocation**	**Metastatic suppressor genes**	**Chromosomal location**	**Metastatic suppressor gene identified in other tumors**
■miR-495	14q32.31	*DLC1*	8p22	Deleted in liver cancer 1, Hepatocellular carcinoma [19]
■miR-18a	13q31.3	*BRMS1*	11q13-q13.2	Breast cancer metastasis suppressor 1; Non-small cell lung cancer [20]
● miR-196a*	12q13.3	*CASP8*	2q33.34	Caspase 8, apoptosis-related cysteine peptidase; Neuroblastoma [21]
● miR-549	15q25.1	*LZTS1*	8p22	Leucine zipper putative tumor suppressor 1, Uveal melanoma [14]
● miR-497*	17p13.3	*KISS1*	1q32.1	KiSS-1 metastasis suppressor; Gastric cancer [22]

■ - miRNAs expressed in non-metastatic melanoma and absent in metastatic melanoma. ● - miRNAs expressed in metastatic melanoma and absent in non-metastatic melanoma.

**Table 3:** Represents the list of oncomirs in uveal melanoma, targeting tumor suppressor genes.

**miRNA**	**Chromosomal location**	**Tumor suppressor genes**	**Chromosomal location**	**Tumor suppressor gene identified in other tumors and disorders**
▀miR-586	6p12.3	*CDKNIC*	11p15.5	Cylin dependent kinase inhibitor 1c, Rhabdoid tumor [23]
▀miR-493*	14q32.1	*PDGFRL*	8p22-p21.3	PDGF receptor beta-like tumor suppressor, Melanoma [24]
▀miR-377	14q32.1	*CAV1*	7q31.1	Caveolin 1, caveolae protein, Lung carcinoma [25]
▀miR-376c	14q32.1	*CDKN1A*	6p21.2	Cyclin dependent kinase inhibitor 1A, Laryngeal squamous cell carcinoma [26]
▀miR-369-3p	14q32.1	*FZD6*	8q22.3-q23.1	Frizzled homolog 6, melanocyte physiology, Uveal melanoma [13]
▀miR-34c-5p	11q23.1	*TGFBR2*	3p22	Tumor growth factor beta receptor 2, Colon cancer [27]
▀miR-26a-2*	12q14.1	*EXLT3*	8p21	Exotoses( multiple) like-3, Bone tumor [28]
▀mir-218	4p15.31	*HYAL1*	3p21.31	Hyaluronoglucosaminidase 1, Head and neck squamous cell carcinoma [29]
▀mir-19b-1*	13q31.3	*RB1*	13q14.2	Retinoblastoma 1 [30]
▀mir-181a*	1q31.3	*BRCA2*	13q12.3	Breast cancer type2 susceptibility protein, Breast cancer [31]
▀mir-154	14q32.31	*NEK1*	4q33	(Never in mitosis gene a)-related kinase 1 [32]
▀miR-133a	18q11.2	*DLEC1*	3p22-p21.3	Deleted in lung and esophagael cancer, Gastrointestinal cancer [33]
▀miR-129*	7q32.1	*TSSC1*	2p25.3	Tumor suppressing subtransferable candidate 1 [34]
▀mir-10a	17q21.32	*LATS1*	6q25.1	Larger tumor suppressor , homolog1 [35]
▀mir-1	1q13.33	*PINX1*	8p23.1	PIN2 –interacting protein 1 [36]
▀Let-7e	19q13.33	*GAS1*	9q21.3-q22	Growth arrest specific 1, Melanoma [37]
●mir-885-5p	3p25.3	*PDGFRL*	8p22-p21.3	Platelet–derived growth factor beta like tumor suppressor [24]
●miR-585	5q35.1	*RPS29*	14q22.1	Ribosomal protein S29, colorectal cancer [38]
●mir-640	19p13.41	*S100A2*	2q14.3	S100 calcium binding protein A2, Head and neck cancer [39]
●miR-512-5p	19q13.41	*BIN-1*	2q14.3	Bridging integrator, prostate cancer [40]
●mir-556-5p	1q23.3	*TP53*	17p13.1	Tumor suppressor protein P53, Osteoscarcoma [41]
●mir-135b*	1q32.1	*HYAL1*	3p21.3-p21.2	Hyaluronoglucosaminidase 1, Head and neck squamous cell carcinoma [29]
●mir-325	Xq21.1	*PTPRG*	3p14.2	Protein tyrosine phosphatase receptor type G, Nasopharyngeal carcinoma [42]
●mir-99a*	21q21.1	*SEMA3B*	3p21.31	Sema domain (Ig) short basic domain secreted, Lung cancer [43]
●miR33a	22q13.2	*TSC2*	16p13.3	Tuberous sclerosis protein 2, Lymphangioleiomyomatosis [44]

■ - miRNAs expressed in non-metastatic melanoma and absent in metastatic melanoma. ●- miRNAs expressed in metastatic melanoma and absent in non-metastatic melanoma.

## Discussion

In the field of Immuno histochemistry (IHC) and FISH, it is routine clinical practice to analyze tumor status in reference to chromosomal aberration. IHC is applied to identify over expression of proteins, yet it also involves inter observer variability and leads to false positive results. FISH is an accurate and sensitive method to study gene amplification studies but needs an expensive fluorescence microscope and the signal fades in course of time. CISH is a cytogenetic study, detecting aneuploidy in both interphase and metaphase within a tumor section.

Interpretation difficulties were encountered occasionally with overlapping nuclei and particulate debris on slides. Heavily pigmented tissues that were not completely depigmented by either method were not taken into account due to signal interference by pigments. CISH reveals chromosomal abnormalities in the given sample but not the genes that are present in the deleted region. Deleted regions are believed to harbor tumor suppressor genes. Non-random chromosomal abnormalities in uveal melanoma linked to metastatic death is yet to be established as effective clinical predictive testing tool. Gene expression analysis identifies deregulated genes in the given tumor samples. MicroRNAs are endogenous molecules that play a significant role in gene regulation.

As our aim was to analyze aberrations in chromosome 1, 3, and 8 in uveal melanoma, an in depth study on intra tumor heterogeneity was not performed. Other cytogenetic studies in uveal melanoma have revealed the presence of monosomy 3 in epithelioid cells and not in spindle cells. Studies on genetic heterogeneity in uveal melanoma has been reported in monosomy 3 in spindle, epithelioid and mixed cell type. Though tumor biopsies can be used for diagnosis, prognosis, and therapeutic decision making, a better insight in identifying patients with high risk could be materialized by assessing samples from several other areas of tumor by the transscleral/transvitreal approach [45].

Studies have reported the presence of miRNAs in cell lines, primary cultured melanomas. Worley et.al have identified differentially expressed miRNAs that could act as prognostic biomarkers of metastatic risk compared to primary uveal melanoma with low metastatic risk [16]. They have identified 6 miRNAs discriminators that accurately that accurately distinguish class I (low metastatic risk) and class II (high metastatic risk). To our knowledge there are no published studies on miRNA expression profiling in formalin fixed paraffin embedded sections in uveal melanoma. MicroRNAs from formalin fixed paraffin embedded sections (FFPE) show reliable expression level compared to snap frozen samples, though larger number of FFPE cells are required to isolate total RNA compared to snap frozen cells [46].

We have studied miRNA expression profiling on tumors classified as non invasive and liver metastasis as per case registry and presence of monosomy in biological replicates. Nineteen miRNAs were found to be expressed only in class I tumors and not in class II and 11 miRNAs were found to be expressed only in class II and not in class I. Similar scenario was observed in Worley et al study. Similarly miRNA expression is found to be lower in cancer cells compared to normal cells. The selected tumors were found to harbor oncomirs in both choroidal melanoma and metastasizing melanoma targeting metastatic suppressor genes (Table 2) and tumor suppressor genes (Table 3).

None of the differentially expressed miRNAs in either case were found to be located on the chromosomes which have been proved to carry chromosomal abnormalities. Rather it was found that the differentially expressed miRNAs bind to 3’UTR segments of genes that were often found to be deleted in chromosomal regions 8p22, 13q, and 17p in uveal melanoma (Table 2 and Table 3). Prediction targets of has-miR-196a, a metastatic suppressor gene-CASP8 observed in neuroblastoma cells. Repression of caspase-8 reduces metastasis without affecting the primary tumor. Martinez et al. [46] have observed that hypermethylation and silencing of CASP8 is correlated to glioblastoma multiforme.

Predicted target DLC1, deleted in liver cancer, is found to act as tumor suppressor in hepatocellular and non-small cell lung carcinoma [47]. In our target prediction study, we have observed that miR-495 binds to 3’UTR segment of DLC1 and metastatic suppressor gene in breast carcinoma call line M4A4 [48]. miR-18a predicted target BRMS1, a nuclear metastasis suppressor gene, is found to suppress experimental metastasis of melanoma, bladder and ovarian carcinomas [49]. *KISS1*, a cytoplasmic metastasis suppressor gene is targeted by miR-497. miR-556-5p targets tumor protein P53 and miR-19b1 targets retinoblastoma associated protein. miR-155 an upregulated miRNA in breast, lung and colon cancer is found to be upregulated in both the tumors.

These results indicate the need for further functional studies to validate the presence of oncomirs in metastatic and non-metastatic uveal melanomas that target metastatic suppressor genes and tumor suppressor genes. Identification of these genes will explore the significant miRNAs that play a major role as biomarkers in tumor progression and metastasis.
